# Treatment Guidelines for Rare, Early-Onset, Treatment-Resistant Epileptic Conditions: A Literature Review on Dravet Syndrome, Lennox-Gastaut Syndrome and CDKL5 Deficiency Disorder

**DOI:** 10.3389/fneur.2021.734612

**Published:** 2021-10-25

**Authors:** Richard F. Chin, Ana Mingorance, Benjamin Ruban-Fell, Isabelle Newell, Jenni Evans, Kishan Vyas, Charlotte Nortvedt, Sam Amin

**Affiliations:** ^1^Royal Hospital for Sick Children, Edinburgh, United Kingdom; ^2^The Loulou Foundation, London, United Kingdom; ^3^Dracaena Consulting, Madrid, Spain; ^4^Costello Medical, London, United Kingdom; ^5^Costello Medical, Cambridge, United Kingdom; ^6^GW Pharma Ltd., London, United Kingdom; ^7^Bristol Royal Hospital for Children, Bristol, United Kingdom

**Keywords:** epilepsy, treatment, literature review, rare disorders, guidelines, CDKL5 deficiency disorder, Dravet syndrome, Lennox-Gastaut syndrome

## Abstract

**Background:** Dravet syndrome (DS), Lennox-Gastaut syndrome (LGS) and CDKL5 deficiency disorder (CDD) are rare epileptic conditions, characterised by drug-resistant seizures. Seizure management in these patients requires careful therapy selection. This targeted literature review (TLR) aimed to collate and synthesise information from country-specific and international treatment guidelines for DS, LGS and CDD.

**Methods:** A TLR was performed between 25^th^ January and 11^th^ March 2021. Online rare diseases and guideline databases were manually searched in addition to websites of national health technology assessment bodies for the following countries: Australia, Canada, France, Germany, Israel, Italy, Japan, Spain, Switzerland, UK and US, as defined by pre-specified eligibility criteria. Search terms, developed for each condition, were translated into local languages where appropriate. Descriptive analyses were performed to examine the geographical distribution of included guidelines; methodologies used to develop guidelines; cross-referencing of treatment recommendations made within other guidelines; patterns of treatment recommendations. An author map was created using R version 3.5.1, to visualise the extent of collaboration between authors.

**Results:** Forty total guidelines were included, of which 29, 34 and 0 contained recommendations for DS, LGS and CDD, respectively (some provided recommendations for ≥1 condition). Most were country-specific, with guideline authors predominantly publishing in regional groups. Five guidelines were classified as “International” and displayed connections between author groups in the US, UK, France and Italy. Reported guideline development processes were lacking [43% (17 guidelines) had unclear/absent literature review methodologies] and those reported were variable, including both systematic and targeted literature reviews. Use of expert consultation was also variable. A high degree of heterogeneity was observed in the availability of treatment recommendations across disorders, with 271 and 190 recommendations for LGS and DS, respectively, and contradictory positive and negative treatment recommendations for several drugs in each indication [35% (11/31) and 22% (6/27) in LGS and DS, respectively].

**Conclusions:** This review highlights the need for further high-quality international consensus-based treatment guidelines for LGS, DS, and particularly for CDD (for which no treatment guidelines were identified). Supra-national consensus guidance based on findings from a wider geographical range may improve resource allocation and establish an improved world-wide standard of care.

## Introduction

Dravet syndrome (DS) and Lennox-Gastaut syndrome (LGS) are severe, treatment-resistant developmental epileptic encephalopathies (DEEs), in which seizure activity is associated with general cerebral dysfunction (1). CDKL5 deficiency disorder (CDD) is a more recently-described DEE caused by mutations in the *CDKL5* gene (2–4). Despite their distinct aetiologies, these disorders all feature the onset of seizures in early childhood, as well as severe cognitive and behavioural impairments (1, 5, 6). It is important to manage seizures carefully to avoid injuries, disability, and reduce the risk of life-threatening complications, such as sudden unexpected death in epilepsy (SUDEP) and status epilepticus (SE) (7, 8).

Management of epileptic seizures requires careful therapy selection to optimise seizure control and improve a patient's quality of life (QoL) (9), balanced against significant side effects that are associated with many pharmacological treatments. The three main forms of treatment available are anti-seizure medications (ASMs), dietary modification (typically the ketogenic diet), and surgical intervention (4, 5, 7), with preventative ASMs remaining the mainstay of epilepsy treatment (10).

The management of seizures in patients with DS, LGS and CDD is particularly challenging as the seizures are frequently treatment-resistant (requiring the use of two or more appropriately chosen ASMs), and patients often fail to achieve complete seizure control (4, 7, 9, 11). In addition, therapy with specific mechanisms of action may be required for certain seizure types, and individual responses to these drugs can be variable (5). In some cases, ASMs may also become less effective over time and can even worsen seizure control (5). Physicians must also consider that seizure patterns and progression of these disorders may change over time (9).

Due to the challenges associated with the selection of appropriate ASMs to manage seizures in patients with DS, LGS and CDD, the development and use of treatment guidelines helps to optimise management of these conditions and align best practises and care in both national and international contexts (12). Additionally, the content of such guidelines may be used to inform health technology assessment (HTA) recommendations and play a decisive role in treatment licencing (13, 14). It is therefore widely accepted that treatment guidelines should be developed using robust methods of evidence generation, such as systematic literature reviews (SLRs) and rigorous forms of expert consensus (12). In addition, expert collaboration and the co-ordinated development of guidelines prevent the duplication of efforts and allow the generation of high-quality recommendations, based on learnings from across the globe (15, 16). Whilst these are the ideal considerations, they are not always met, particularly for rare diseases.

Treatment guidelines for rare diseases are often scarce, geography-specific, and are of varying quality largely due to a paucity of high certainty evidence (17, 18). Physicians, support groups and carers of people with rare diseases often need to keep updated with developments in the field; however, clinicians and families may not have the time to collate and analyse available data, and therefore require guidelines to ensure patients receive optimal care (19). In a user satisfaction survey undertaken by the Orphanet website (an online resource which aims to provide high-quality information on rare diseases to a variety of stakeholders), respondents were reported as being interested in accessing more clinical guidelines and review articles than were already available, as well as expanding access to resources from a wider range of countries, highlighting the continued need for robust treatment guidelines (20).

The objective of this targeted literature review (TLR) was to perform a descriptive analysis of available treatment guidelines for the management of DS, LGS and CDD. More specifically, we aimed to:

1. Determine the availability of country-specific and international treatment guidelines for DS, LGS and CDD;
2. Describe the methodology used to develop individual existing guidelines;
3. Assess the extent of collaboration between authors through the identification of shared authors between the included guidelines; and
4. Report the frequency and patterns of existing treatment recommendations for DS, LGS and CDD.

## Methods

### Search Strategy

A TLR was performed between 25^th^ January and 11^th^ March 2021; online information sources were manually searched in accordance with pre-specified search criteria, to identify relevant treatment guidelines. The search strategies used for each information source, and the dates of searches are summarised in Supplementary Table 1.

The search strategy included searches of the following sources: Google, Guideline Central, Orphanet, National Organisation for Rare Disorders (NORD), American Academy of Neurology (AAN), American Epilepsy Society (AES) and International League Against Epilepsy (ILAE). Websites of national HTA bodies for the following countries were also searched: Australia, Canada, France, Germany, Israel, Italy, Japan, Spain, Switzerland, United Kingdom (UK), and United States (US).

Each database was queried with search terms appropriate for its search functionality (e.g., Boolean operators were used where possible) and the specificity of the database (e.g., whether it was a repository of treatment guidelines, in which case search terms for “guidelines” were unnecessary); searches were filtered for guidelines where possible. Search terms included combinations of free-text and terms for each of the indications of interest. These terms were translated into the relevant language where applicable.

### Review Process

Each record identified through the searches was screened for eligibility according to criteria defined using a PICOS (Population, Intervention, Comparators, Outcomes, Study design) approach, as presented in Table 1. Briefly, eligible publications were guidelines or guidance reporting routine pharmacological management of seizures in patients with DS, LGS or CDD in the countries of interest described previously. Eligible publications were classified as “International” if they were developed either for multiple countries or did not specify to which countries they pertained. Guidance or guidelines were defined as publications which were informed by rigorous methods, such as an SLR, had multiple authors or explicitly stated that certain treatments were “recommended”. In addition to guidelines produced by HTA bodies, the review also captured technology appraisal guidance following any conducted technology assessments. Search results were screened by a single reviewer. Where the applicability of the inclusion criteria was unclear, the record was assessed by a second reviewer. Where possible, reviewers who were either fluent or had a high level of proficiency in a relevant language were responsible for the identification, screening and extraction of any guideline documents not published in the English language. For languages in which reviewers were not proficient, the online translation software, DeepL®, was used.

**Table 1:** Eligibility criteria.

**Modified PICOS domain**	**Inclusion criteria**	**Exclusion criteria**
Population	Patients with the following epileptic conditions: •Dravet syndrome •Lennox-Gastaut syndrome •CDKL5 deficiency disorder	Conditions other than those listed
Intervention	Any	None
Outcomes	The document must have discussed the management of the conditions of interest in terms of pharmacological treatment pathways for routine seizure control	•Documents that did not discuss the management in terms of pharmacological treatment pathways •Emergency medication and surgical guidelines
Publication type	Guidelines or guidance documents	Publications other than guidelines
Other considerations	Specifically produced for use in: •EU5 countries (UK, Germany, Spain, Italy, France) •Japan •Australia •Switzerland •Israel •US •Canada	Produced specifically for use in countries that were not of interest
	International guidelines (i.e., guidelines produced for multiple countries that included or potentially included the countries of interest, or guidelines that did not specify which countries they pertained to)	

*EU, European Union; PICOS, Population, Intervention, Comparators, Outcomes, Study design; UK, United Kingdom; US, United States*.

### Data Extraction and Analyses

Guidelines presenting relevant data were extracted into a pre-defined extraction grid. Information extracted for each guideline included: publication date and planned revision date; the organisation that developed the guideline; author names and author affiliations; the methodology used for the development of guidelines, including use of literature reviews and expert consultation; population(s) addressed; pharmacological recommendations by treatment stage and seizure subtype and references to other guidelines, HTA assessments/regulatory body decisions and compiled literature sources (including SLRs, meta-analyses and electronic databases).

Descriptive analyses were performed in Microsoft Excel® to examine: the distribution of identified guidelines across the countries of interest; the methodologies used to develop the treatment guidelines; and the cross-referencing of treatment recommendations made within other guidelines.

The authors involved in developing each of the guidelines identified in this study (including guidelines for both DS and LGS) were mapped into a network, using R version 3.5.1 to visualise whether authors were contributing to >1 guideline and if so, to measure the extent of collaboration between these authors, both on a national and international level.

In order to assess the patterns of positive and negative pharmacological treatment recommendations for each indication, further descriptive analyses were performed. A positive recommendation was defined as an individual ASM that was recommended for use in a specific indication, irrespective of the line of treatment (e.g., first-line) or whether the treatment was adjunctive; whilst a negative recommendation was defined as an individual ASM treatment that was highlighted as a potential option by a guideline but whose use was recommended against (for any reason) in a specific indication, irrespective of the line of treatment or whether the treatment was adjunctive.

## Results

### Characteristics of Included Guidelines

**Figure 1:**
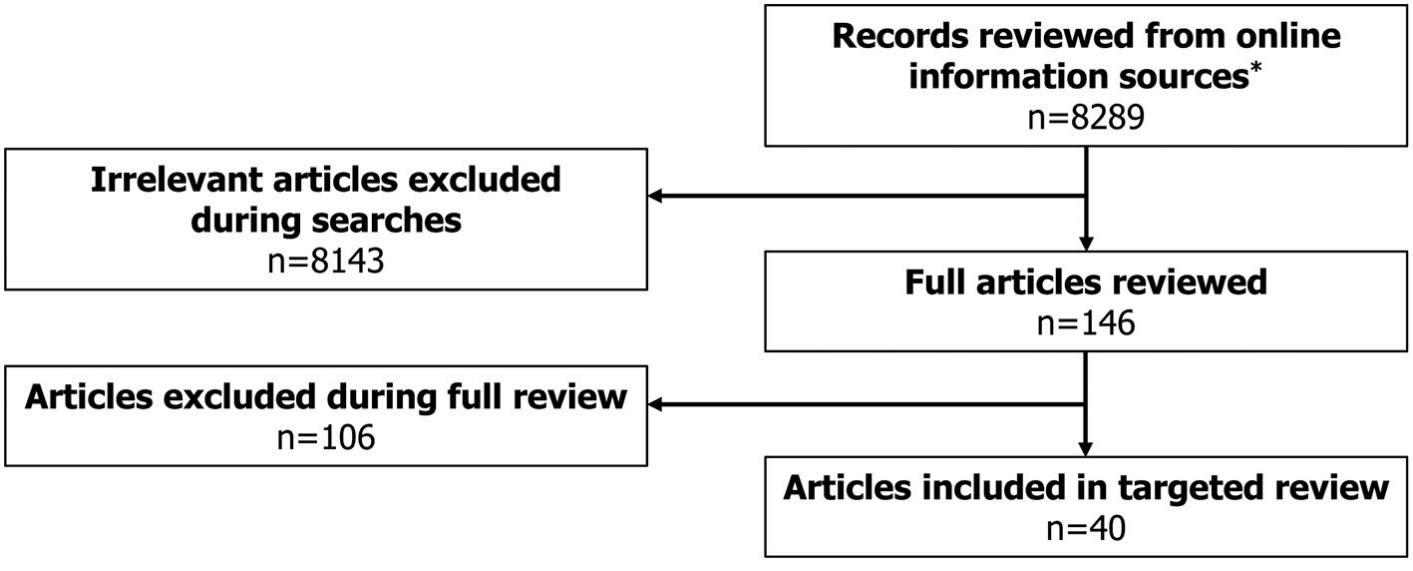
Literature review flowchart. *Online information sources included: Guideline Central, National Organization for Rare Disorders (NORD), American Academy of Neurology (AAN), American Epilepsy Society (AES), International League Against Epilepsy (ILAE), Orphanet, Google, National Institute for Health and Care Excellence (NICE), Pharmaceutical Benefits Scheme (PBS), Canadian Agency for Drugs and Technologies in Health (CADTH), Ministerio de Sanidad, Consumo y Bienestar Social (MSCBS), Agenzia Italiana del Farmaco (AIFA), Haute Autorité de Santé (HAS), Gemeinsamer Bundesausschuss (G-BA), Bundesamt für Gesundheit (BAG), State of Israel – Ministry of Health, Ministry of Health, Labour and Welfare (MHLW).

**Figure 2:**
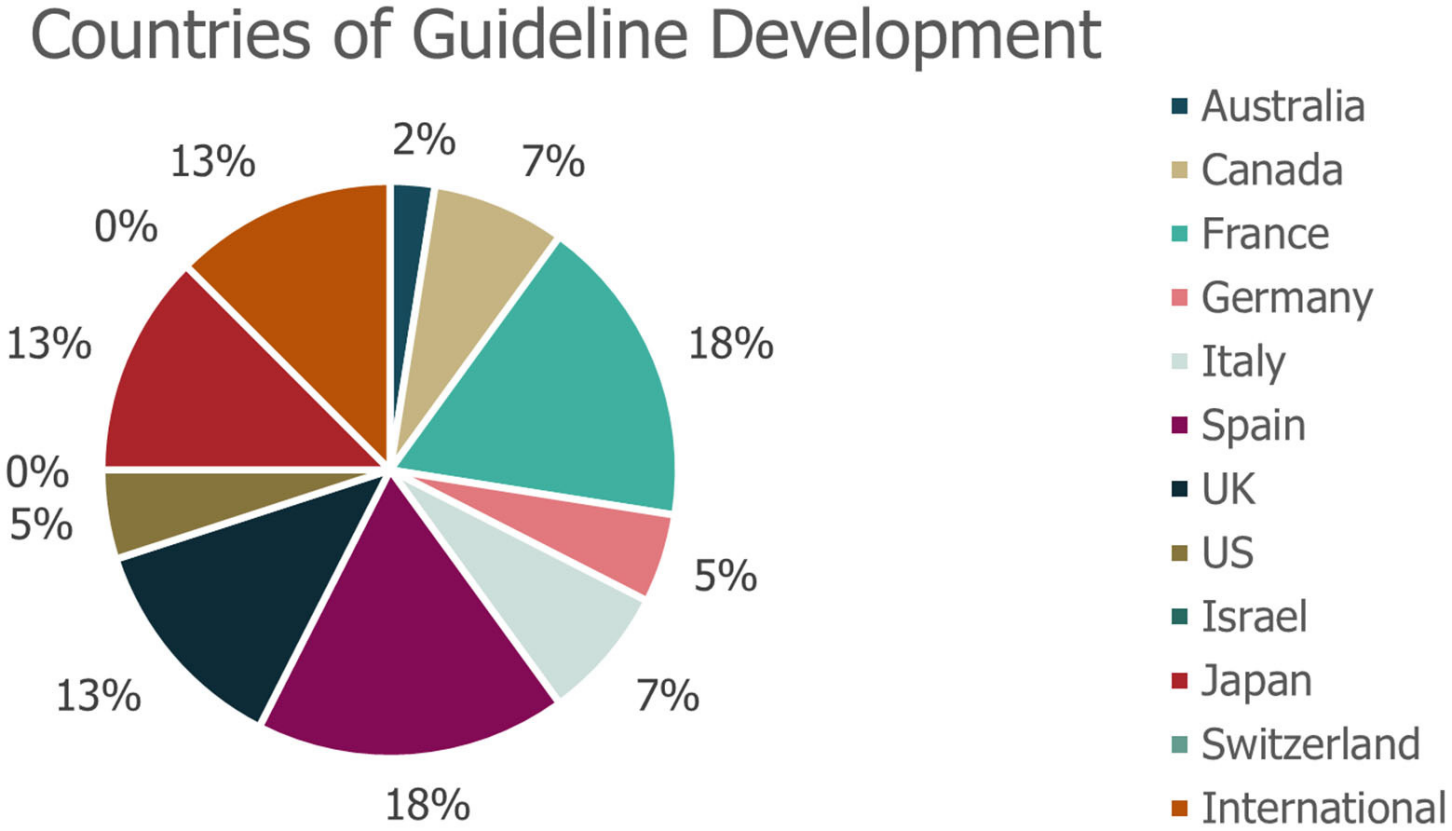
Geographies of identified guidelines. *No guidelines were identified for use in Israel or Switzerland. The geography of guideline use refers to the country for which that the guidance was specifically developed.

A total of 40 eligible records were included in the review (Figure 1), with publication dates ranging between November 2005 and January 2021. More detailed information regarding each of the guidelines is presented in Supplementary Table 2. The majority of guidelines were country-specific (with recommendations intended for patients in a specific country); however, five guidelines were classified as “International” (Figure 2). The countries with the highest number of identified guidelines were France (7; 18%), Spain (7; 18%), Japan (5; 13%) and the UK (5; 13%). No national guidelines were identified for use in Israel or Switzerland. Only three guidelines were identified that developed recommendations specifically for DS or LGS (one in LGS from Germany, one in LGS from an international author group and one in DS from an international author group). The remaining guidelines including recommendations for DS or LGS were identified within broader epilepsy guidelines. Several guidelines were specifically developed for regions within one of the countries of interest (13% [5/40]). Out of these, two UK guidelines were created for use in Scotland, an Italian guideline was developed for the region of Tuscany and two of the seven Spanish guidelines identified were created specifically for the region of Andalusia. None of the guidelines identified were for use in the US at the state level.

### Evidence Base and Methodology for Guideline Development

**Figure 3:**
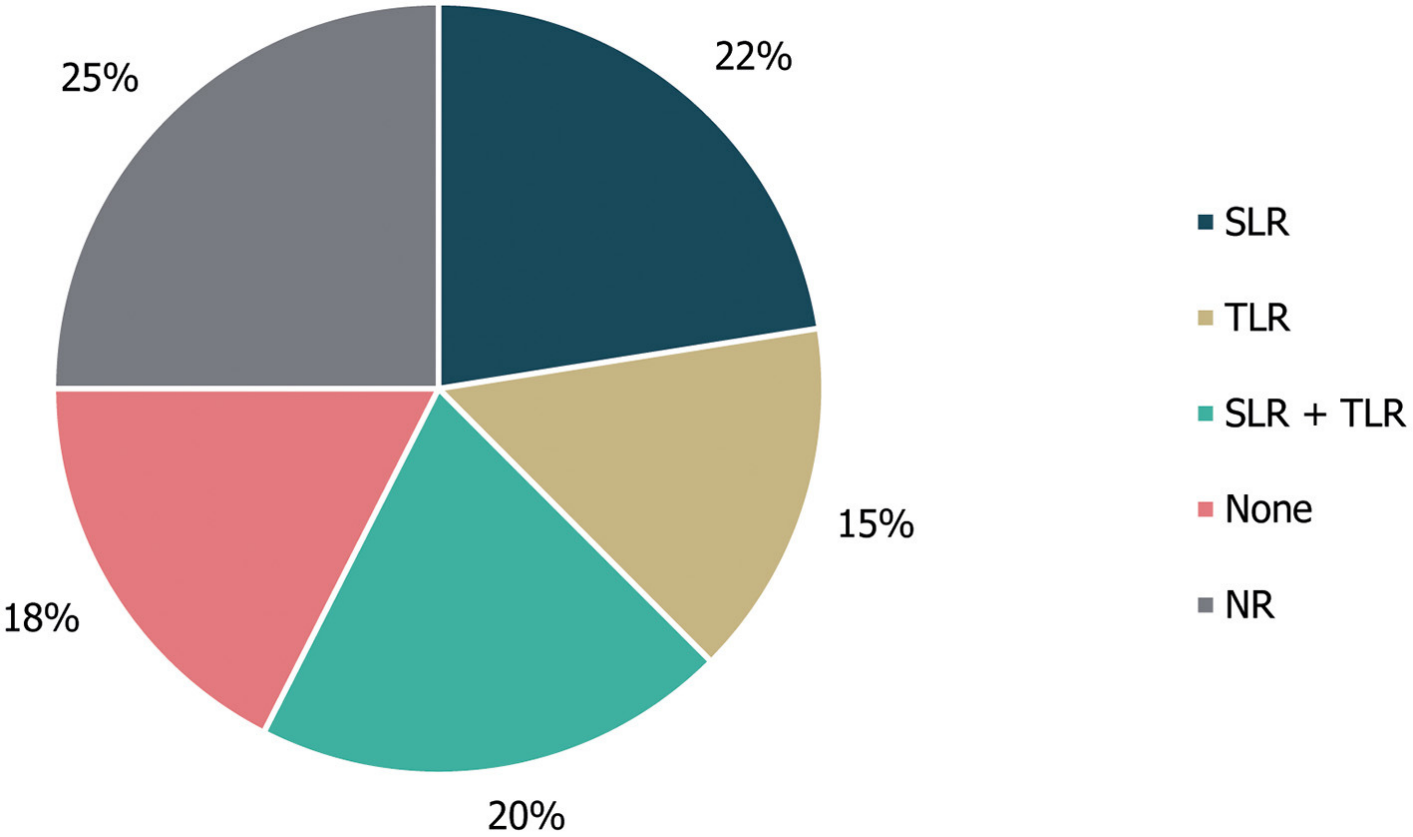
Types of literature review performed to inform guideline development. “None” refers to guidelines in which a literature review was explicitly not used; NR, not reported; SLR, systematic literature review; TLR, targeted literature review.

**Figure 4:**
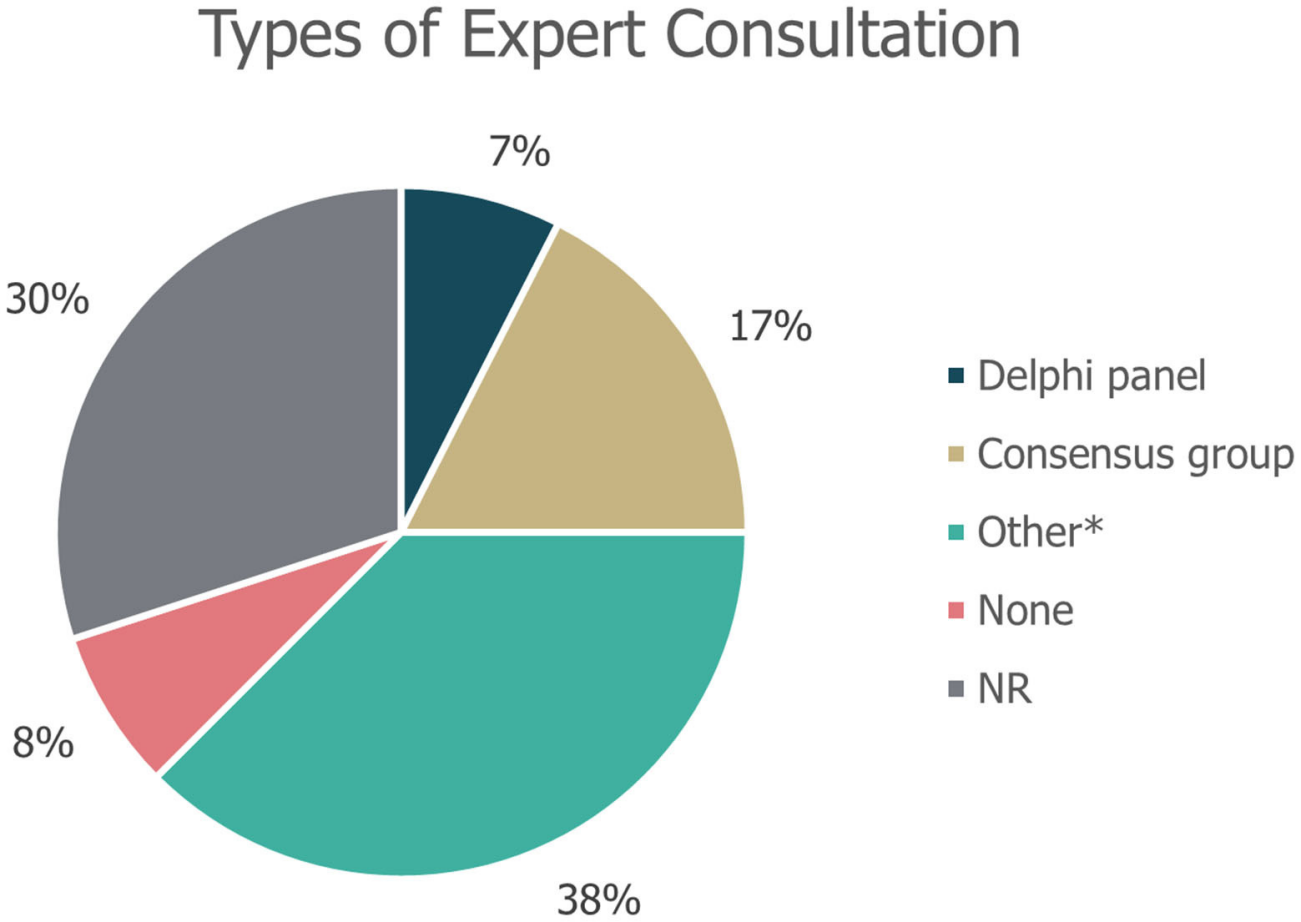
Types of expert consultation performed to inform guideline development. “None” refers to guidelines in which expert consultation was explicitly not used; NR, not reported. *Other refers to working groups or targeted expert interviews.

Of the 40 guidelines identified, 10 (25%) did not specify whether literature reviews were used to inform guideline development. An additional seven guidelines (18%) explicitly stated that a literature review was not used as part of the development process. The remaining guidance documents involved either systematic [22% (9/40)] or targeted [15% (6/40)] literature searches, or a combination of these [20% (8/40)]; (Figure 3). Details on expert consultation were not reported by 12/40 guidelines (30%); three guidelines (8%) explicitly did not include any form of expert consultation. Only three guidelines (7%) involved a Delphi panel to inform guidance, while seven guidelines (17%) were based on formal consensus group exercises; the remaining 15 guidelines (38%) utilised other forms of expert consultation, such as working groups or targeted expert interviews (Figure 4). Although 20/40 (50%) of guidelines reported the use of a combined development approach consisting of a literature review and expert consultation, only one of the guidelines explicitly used an SLR and Delphi panel in combination.

**Figure 5:**
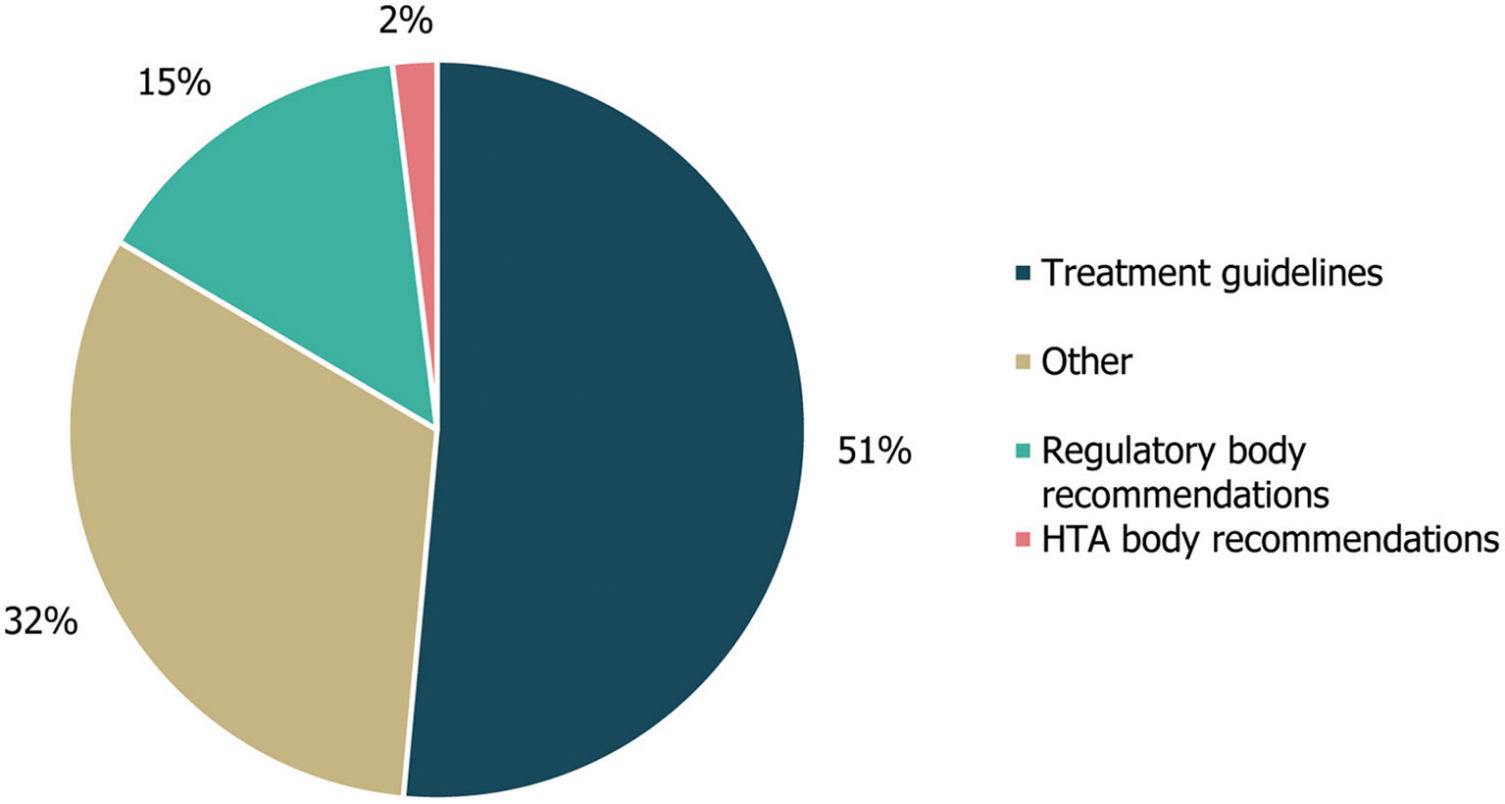
Guideline cross-referencing to other treatment guidelines and regulatory/HTA recommendations. Cross-referencing refers to the number of different treatment guidelines, regulatory body recommendations, HTA body recommendations or other references that were cited within the guidelines identified in this study, either in the body of the guideline text or in accompanying reference lists. “Other” references included a Cochrane systematic literature review, an information website, a narrative review and a consensus conference report. HTA, health technology assessment.

A review of cross-referencing between the included guidelines and other published guidance/literature reviews revealed that citations within the identified guidelines mainly referenced other treatment guidelines (53/103; 51%) or other compiled literature sources (33; 32%), with the majority of the latter consisting of SLRs included in the Cochrane Database of Systematic Reviews (20/33; Figure 5). Citations also referenced 15 (15%) regulatory body recommendations, two of which were made to HTA body recommendations. The three documents most frequently referenced (ten, six and seven times, respectively) were the UK's National Institute for Health and Care Excellence (NICE)'s guidance on the diagnosis and management of epilepsies (CG137) (21), an SLR from the Cochrane Database of Systematic Reviews on the treatment of infantile spasms (22) and a systematic literature review from the Cochrane Database of Systematic Reviews on the treatment of Lennox-Gastaut syndrome (23, 24).

### Extent of Author Collaboration

**Figure 6:**
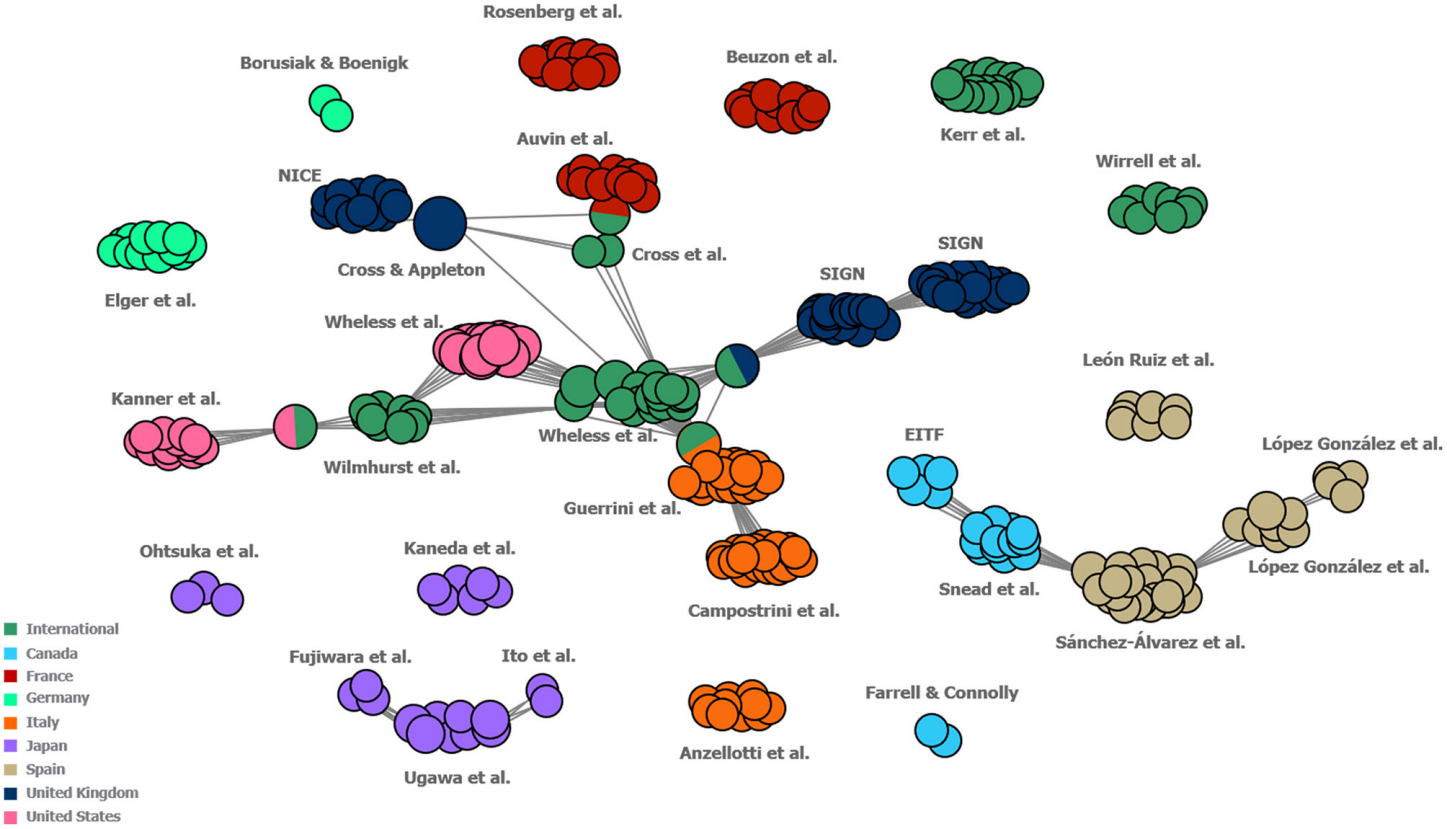
Map of collaboration between the author groups of included guidelines. Each individual circle represents one author of a guideline. Each “cluster” represents the group of authors that developed one guideline. Each cluster is labelled with the names of its respective first author(s). Guidelines which share one or more authors between them are connected by grey lines, with single circles between guideline clusters representing the individuals who authored both guidelines in question. Guidelines were classified as “International” if they were developed either for multiple countries or did not specify to which countries they pertained. Guidelines for which author names were not reported have not been included in this figure. EITF, Epilepsy Implementation Task Force; NICE, National Institute for Health and Clinical Excellence; SIGN, Scottish Intercollegiate Guidelines Network.

In the author map, which was developed to investigate the extent of national and international levels of collaboration by visualising a network of the authors involved in developing each of the guidelines identified in this study (including guidelines developed for both DS and LGS), connections were identified between international treatment guidelines and US, UK, French and Italian guideline author groups as well as between Canadian and Spanish guideline author groups. Other regional guidelines displayed only occasional connections between author groups within the region in question (these were mostly found to be within the Japanese region; Figure 6).

### Treatment Recommendations for Dravet Syndrome

**Figure 7:**
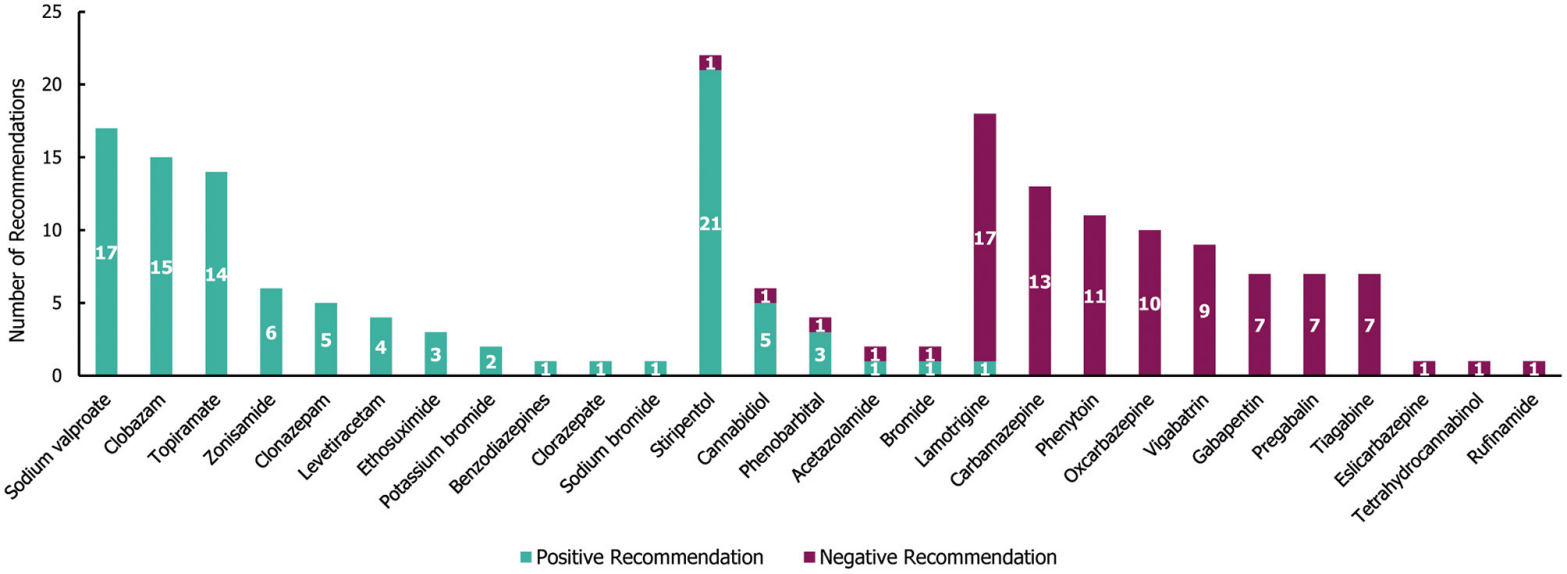
Treatment recommendations for Dravet syndrome. *N* = 190 (101 positive and 89 negative recommendations) from 29 guidelines. Positive recommendation: use of an individual ASM that was recommended for use in a specific indication, irrespective of the line of treatment (e.g., first line) or whether the treatment was adjunctive; Negative recommendation: an individual ASM treatment that was highlighted as a potential option by a guideline but whose use was recommended against (for any reason) in a specific indication, irrespective of the line of treatment or whether the treatment was adjunctive.

In the 29 guidelines identified for DS, a total of 190 individual treatment recommendations were made (irrespective of the line of treatment; Figure 7). Of these treatment recommendations, similar proportions were positive (53%; 101/190) and negative (47%; 89/190). Most of the recommended treatments (21/27) received either exclusively negative or positive recommendations, with only stiripentol, cannabidiol, phenobarbital, acetazolamide, bromide, and lamotrigine having received both (Figure 7). Out of the 27 treatments, 11 received exclusively positive recommendations for use in DS, of which sodium valproate, clobazam and topiramate had the highest number (≥14 each). However, stiripentol had the highest number of positive recommendations (21), as well as one negative recommendation. Of these, only stiripentol and cannabidiol have been approved by the Food and Drug Administration (FDA) and European Medicines Agency (EMA) for the treatment of seizures in Dravet syndrome; both drugs received a negative recommendation due to not being licenced in the region of interest at the time of guideline publication (25–28). A number of treatments (10/27) received exclusively negative recommendations for use in DS, of which carbamazepine, phenytoin, oxcarbazepine and vigabatrin had the highest number (≥9 each).

Out of the 101 total positive treatment recommendations for DS, 37 (37%) were recommended for a specific line of treatment (18 for first-line, 19 for second line; see Supplementary Table 3). Sodium valproate received the highest number of positive first-line recommendations (ten), followed by topiramate (five) and stiripentol (two; approved only as an add-on therapy to sodium valproate and clobazam) (29). Clobazam received the highest number of positive second-line recommendations (four). There were only three seizure type-specific recommendations for DS, two of which were positive recommendations for the use of stiripentol in tonic-clonic seizures, and one was a negative recommendation for the use of lamotrigine in myoclonic seizures.

### Treatment Recommendations for Lennox-Gastaut Syndrome

**Figure 8:**
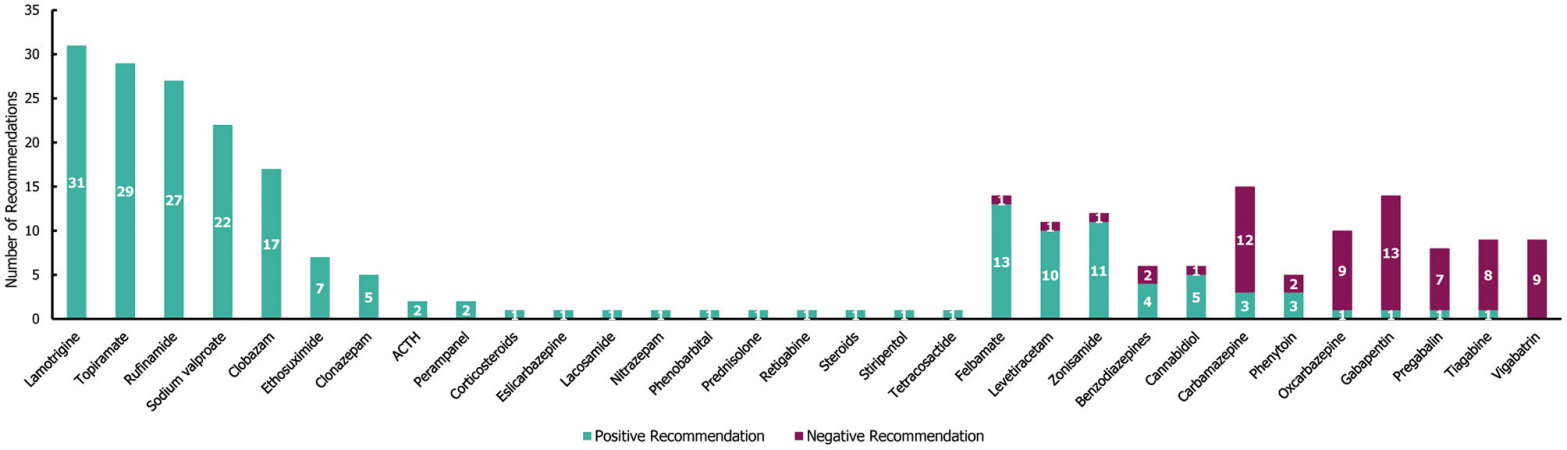
Treatment recommendations for Lennox-Gastaut syndrome. *N* = 271 (205 positive and 66 negative treatment recommendations) from 34 guidelines. Positive recommendation: use of an individual ASM treatment that was recommended for use in a specific indication, irrespective of the line of treatment (e.g., first line) or whether the treatment was adjunctive; negative recommendation: an individual ASM treatment that was highlighted as a potential option by a guideline but whose use was recommended against (for any reason) in a specific indication, irrespective of the line of treatment, or whether the treatment was adjunctive.

In the 34 guidelines identified for LGS, a total of 271 individual treatment recommendations were made irrespective of line of treatment (Figure 8). Of these 271 individual recommendations, 205 (76%) were positive and 66 (24%) were negative. Nearly two-thirds of the drugs that were recommended (65% [20/31]) received either exclusively negative or positive (1 and 19 drugs, respectively) recommendations for LGS. However, 35% (11/31) of drugs received both negative and positive recommendations. Of the 19 drugs that received positive recommendations for use in patients with LGS; lamotrigine, topiramate and rufinamide received the most (with ≥27 positive recommendations each, and no negative recommendations; Figure 8). These three drugs have been specifically approved for the treatment of epilepsy in LGS (30–32) in addition to felbamate (13 positive and one negative recommendation), clobazam (17 positive recommendations) and cannabidiol (5 positive and one negative recommendation) (28, 33, 34). Vigabatrin was the only drug with exclusively negative recommendations for the treatment of LGS (nine in total). Carbamazepine and gabapentin received the highest number of individual negative recommendations (receiving 12 and 13 negative recommendations across the guidelines, respectively).

Out of the 205 positive treatment recommendations, 63 (31%) were recommended for a specific treatment line for LGS (Supplementary Table 4). Sodium valproate received the highest number of positive recommendations as a first-line therapy (14), whereas lamotrigine received the highest number of positive recommendations as a second-line therapy (9). All negative recommendations for a specific line of treatment (6/66 [9%]) were associated with second-line treatment recommendations (with carbamazepine, gabapentin, oxcarbazepine, pregabalin, tiagabine and vigabatrin receiving one each). Additionally, there were 40 seizure type-specific recommendations for LGS (35 positive, 5 negative), which covered a wide range of seizure types, including absence, atonic, atypical absence, crisis episode, generalised, myoclonic, tonic, tonic-atonic and tonic-clonic (although most seizure-type specific recommendations were only made once among the guidelines). The two most frequent seizure type-specific recommendations (each receiving 3) were positive recommendations for ethosuximide in atypical absence seizures and topiramate in atonic seizures.

### Treatment Recommendations for CDKL5 Deficiency Disorder

Although there are publications that describe treatment response to specific drugs or diets in patients with CDD (35), no treatment guidelines for the management of routine seizures in CDD were identified.

## Discussion

This review provides a comprehensive overview of available guidelines and their treatment recommendations for DS, LGS and CDD in 11 countries across Europe, North America and Asia Pacific. The main findings were: whilst there were guidelines for DS and LGS, none were identified for CDD; there were relatively few international treatment guidelines, and in particular, very few that specialised specifically in DS or LGS (most recommendations for DS or LGS were identified within broader epilepsy guidelines); a wide variety of methodologies were used in guideline development; there was limited collaboration between author groups outside of Europe and North America; and a lack of homogeneous treatment recommendations. Most guidelines were country-specific (five guidelines were classified as “International;” two and three of which reported recommendations for DS and LGS, respectively), and five guidelines were specifically developed for a particular region within a given country, which may be reflective of differing drug availabilities in a given country or region.

Key links were identified between the author groups of two international guidelines (36, 37) and guidelines from the US (38), UK (SIGN) (39), Italy (40), and France (41). Additionally, a separate link was observed between the author groups of two Canadian guidelines (42, 43) and Spanish groups (44). This suggests a reasonably well-defined network between North America and Europe, whilst highlighting a lack of collaboration between the author groups in North America, Europe and Japan. Although several of the guidelines were apparently developed in regional groups, with no connections to other guideline author groups identified in the author map (particularly those developed for Germany and Japan), there were no major divergences observed in the recommendations across the geographies. Unsurprisingly, there was a lack of guidelines developed specifically for either LGS or DS (3), and of these, all were developed by international author groups (45–47). Despite the general consensus observed among the included guidelines, bringing together national expert groups and corresponding pooling of clinical expertise, for example via supra-national bodies, could still be beneficial for the development of internationally valid and relevant guidance specifically for these conditions, and in particular, for CDD. For rare conditions with limited high-quality clinical trial data, international consensus recommendations from clinical experts offer a globally accepted standard of care, to which clinicians worldwide can refer (48). This is of particular benefit in regions where no national guidance is available.

For example, in 2013, the International League Against Epilepsy (ILAE) developed a report investigating the efficacy of ASMs as monotherapies for untreated epilepsy (49). Similar international guidance for treatment of DS, LGS and CDD could provide much needed guidance in a global context, accepting that implementation would depend on local infrastructure, resource availability or the healthcare systems in place (50).

The current TLR highlighted the wide variety of methodologies used to develop treatment guidelines. Just over half of the guidelines specified their development process in relation to literature reviews [58% (23/40)] and approximately two-thirds specified some type of expert consultation [65% (25/40)] but reporting of methodology overall was unclear or absent in many instances, and only one guideline used a combination of an SLR and Delphi panel, which is considered to be the “gold standard” of guideline development. SLRs are considered to be the most robust methodology for evidence synthesis, and Delphi panels are recommended for use in healthcare settings as a reliable means of determining consensus for defined clinical problems (51–53). A lack of the combined approach of an SLR and Delphi panel for guideline development highlights a need for standardisation in guideline development and reporting, for which tools to facilitate the improvement of guideline reporting are currently available (e.g., the AGREE checklist) (54). The frequent references that were made by both UK and non-UK guidelines to recommendations by NICE and Cochrane reviews (21–23) that are widely recognised as using rigorous and high-quality development processes (23, 24), may demonstrate the perceived value of guidelines or reviews with robust methodologies regardless of their intended geographical region of influence. Similarly, only one guideline made reference to the ILAE website, which is an international resource for current and emerging standards and best practise in epilepsy and has collaborated with organisations such as AAN, NICE, and the World Health Organization to outline evidence-based clinical practise guideline development (55).

There were a large number of treatment recommendations made for DS (190) and LGS (271), while no individual treatment recommendations were made for CDD. We infer that these findings reflect the lack of high level evidence for preferred treatments, and the refractory nature of the seizures in each of the three syndromes (4, 7, 56). Further, our results also highlight a lack of treatment guidelines in diseases of more recent clinical description and that have no licenced medications, such as CDD (6). Consequently, there is an urgent need to develop up-to-date treatment guidelines for CDD (4, 57). A lag between completion/publication of clinical research and the incorporation of their key findings into disease specific guidelines is expected, and this is reflected in the absence of identified guidelines for DS that include recommendations for the drug fenfluramine, which is the latest treatment approved for this indication (58, 59). However with the recent emergence of novel treatments for DS, LGS and CDD (3, 59–61), and for other diseases in general, it is hoped that this lag will become as short as possible.

Interestingly, there was continued recommendation for use of older drugs, such as sodium valproate, for treating seizures in DS and LGS. The consistent recommendation of more traditionally used ASMs may indicate a limited pool of available treatment options and the corresponding need for new and effective treatments that target the specific aetiologies of each disorder (62). In addition, many of the treatments that were widely recommended in the review have no licence available for the indications of interest and are instead more generally indicated for the management of seizures. While stiripentol and cannabidiol have been approved by the FDA and EMA as orphan products for the treatment of DS (25–28), other medications that received a high number of positive recommendations for DS are either licenced more generally for the treatment of epilepsy (e.g., sodium valproate) or for specific seizure types (e.g., topiramate) (7, 25, 26, 63, 64). Similarly, whilst topiramate, lamotrigine, felbamate, rufinamide, clobazam and cannabidiol have been approved by the FDA for use in LGS (28, 30–34), a number of other medications that received positive recommendations for LGS were licenced for all forms of epilepsy (e.g., sodium valproate), or for specific seizure types (e.g., zonisamide) (56, 63, 65).

Due to the targeted nature of the review, some limitations were present; eligibility of all records in the analysis was assessed by a single reviewer, with a second adjudicating the decision of whether a guideline was eligible to include when the applicability of the inclusion criteria was unclear. This approach differs slightly from the dual review technique adopted in systematic literature reviews (66). Additionally, this TLR searched less standard sources than those typically seen in a systematic review (e.g., Embase, MEDLINE/PubMed), for example through the use of Google, as well as medical society and guideline developer websites. Given that not all guidelines are published in traditional medical journals, or necessarily in the English language, this approach ensured a focus on sources that specifically orientated towards, indexing guidelines to minimise the risk of missing local guidelines. While less standard for a literature review, these sources were able to return a large number of highly specific records and provided a multinational overview of the available guidelines and their treatment recommendations in the absence of previously conducted analyses. Additionally, the study aimed to provide an overview in a broad sample of countries likely to be highly influential in the development of treatment guidelines. As such, with the focus on Australia, Canada, France, Germany, Israel, Italy, Japan, Spain, Switzerland, the UK and the US only, the results may not fully represent the international landscape of treatment guidelines for DS, LGS and CDD.

The scope of this review was also limited to treatment guidelines for the routine management of seizures with individual ASMs and was not designed to capture publications including guidance on combination therapies, whether a combination of ASMs or ASMs and/or dietary modification and/or surgery, non-pharmacological therapies or rescue therapies used in treating seizures in acute situations. In addition, the review does not capture treatment guidelines published after February 2021 or those that are currently in development. As treatment guidelines are updated after advancements in clinical care and drug approval have been made (67), the individual recommendations in this review should be interpreted in the context and date that they were made (all identified papers were published between November 2005 and January 2021).

The results of this review suggest the need for further high-quality international consensus-based guidance, influenced by a more diverse range of geographical regions, for the treatment of DS, LGS, and especially for CDD (for which no treatment guidelines could be identified). Following recent approvals for these indications, there is a need to reduce the delay between completion of clinical research and the incorporation of their key findings into disease specific guidelines. In addition, the presence of contradictory positive and negative treatment recommendations for many different drugs in each indication, highlights the need for clarification and consensus on evidence-based first- and second-line drugs to treat each disorder. Supra-national consensus guidance would support the development of local treatment guidance, may improve resource allocation and establish an improved international standard of care.

## Data Availability Statement

The original contributions presented in the study are included in the article/Supplementary Material, further inquiries can be directed to the corresponding author/s.

## Author Contributions

BR-F, IN, JE, and KV: substantial contributions to study conception and design. RC, AM, BR-F, IN, JE, KV, CN, and SA: substantial contributions to analysis and interpretation of the data, drafting the article or revising it critically for important intellectual content, and final approval of the version of the article to be published. All authors contributed to the article and approved the submitted version.

## Funding

This study was sponsored by GW Pharmaceuticals. Support for third-party writing assistance for this article was funded by GW Pharmaceuticals in accordance with Good Publication Practice (GPP3) guidelines (http://www.ismpp.org/gpp3).

## Conflict of Interest

The authors declare that this study received funding from GW Pharmaceuticals. The funder had the following involvement in the study: contributions to the conception of the study, interpretation of data for the work, revising the publication critically for important intellectual content, and decision to submit the work for publication. Editorial and medical writing services were provided by Costello Medical. RC has provided consultancy and speaker services, has participated in events and studies, for GW Pharmaceuticals, Eisai, Zogenix, and Neopharm Group, has shares in the Rize Medical Cannabis, and Life Sciences UCITS ETF. AM has received consulting fees from Encoded Therapeutics, F. Hoffmann-La Roche, GW Pharmaceuticals, Neurelis, Ovid Therapeutics, Praxis Precision Medicines, and Xenon Pharmaceuticals. IN, BR-F, and JE employee of Costello Medical. KV and CN employee of GWPharma Ltd.

## Publisher's Note

All claims expressed in this article are solely those of the authors and do not necessarily represent those of their affiliated organizations, or those of the publisher, the editors and the reviewers. Any product that may be evaluated in this article, or claim that may be made by its manufacturer, is not guaranteed or endorsed by the publisher.

## References

[1] Jain P Sharma S Tripathi M. Diagnosis and management of epileptic encephalopathies in children. Epilepsy Res Treat. (2013) 2013:501981. 10.1155/2013/501981 23970964 PMC3736403

[2] Fehr S Wilson M Downs J Williams S Murgia A Sartori S . The CDKL5 disorder is an independent clinical entity associated with early-onset encephalopathy. Eur J Hum Genet. (2013) 21:266–73. 10.1038/ejhg.2012.156 22872100 PMC3573195

[3] Olson HE Demarest ST Pestana-Knight EM Swanson LC Iqbal S Lal D . Cyclin-dependent kinase-like 5 deficiency disorder: clinical review. Pediatr Neurol. (2019) 97:18–25. 10.1016/j.pediatrneurol.2019.02.015 30928302 PMC7120929

[4] National Organisation For Rare Disorders (2015). CDKL5. Available online at: https://rarediseases.org/rare-diseases/cdkl5/ (accessed May 2021).

[5] Asadi-Pooya AA. Lennox-Gastaut syndrome: a comprehensive review. Neurol Sci. (2018) 39:403–14. 10.1007/s10072-017-3188-y 29124439

[6] Jakimiec M Paprocka J Smigiel R. CDKL5 deficiency disorder-a complex epileptic encephalopathy. Brain Sci. (2020) 10:107. 10.3390/brainsci10020107 32079229 PMC7071516

[7] National Organization for Rare Disorders. Dravet Syndrome. (2018). Available online at: https://rarediseases.org/rare-diseases/dravet-syndrome-spectrum/ (accessed May 2021).

[8] Krauss GL Sperling MR. Treating patients with medically resistant epilepsy. Neurol Clin Pract. (2011) 1:14–23. 10.1212/CPJ.0b013e31823d07d1 23634355 PMC3613191

[9] Mitchell JW Seri S Cavanna AE. Pharmacotherapeutic and non-pharmacological options for refractory and difficult-to-treat seizures. J Cent Nerv Syst Dis. (2012) 4:105–15. 10.4137/JCNSD.S8315 23650471 PMC3619658

[10] Brown C. Pharmacological management of epilepsy. Prog Neurol Psychiatry. (2016) 20:27–34. 10.1002/pnp.422

[11] MedlinePlus: Lennox-Gastaut syndrome. Available online at: https://medlineplus.gov/genetics/condition/lennox-gastaut-syndrome/ (accessed October 2021).

[12] Guyatt GH Oxman AD Kunz R Vist GE Falck-Ytter Y Schünemann HJ . What is “quality of evidence” and why is it important to clinicians? BMJ. (2008) 336:995–8. 10.1136/bmj.39490.551019.BE 18456631 PMC2364804

[13] Akehurst RL Abadie E Renaudin N Sarkozy F. Variation in health technology assessment and reimbursement processes in Europe. Value Health. (2017) 20:67–76. 10.1016/j.jval.2016.08.725 28212972

[14] Detela G Lodge A. EU regulatory pathways for ATMPs: standard, accelerated and adaptive pathways to marketing authorisation. Mol Ther Methods Clin Dev. (2019) 13:205–32. 10.1016/j.omtm.2019.01.010 30815512 PMC6378853

[15] Grol R Cluzeau FA Burgers JS. Clinical practice guidelines: towards better quality guidelines and increased international collaboration. Br J Cancer. (2003) 89:S4–8. 10.1038/sj.bjc.6601077 12915896 PMC2753001

[16] Qaseem A Forland F Macbeth F Ollenschlager G Phillips S van der Wees P. Guidelines international network: toward international standards for clinical practice guidelines. Ann Intern Med. (2012) 156:525–31. 10.7326/0003-4819-156-7-201204030-00009 22473437

[17] Pavan S Rommel K Marquina MEM Höhn S Lanneau V Rath A. Clinical practice guidelines for rare diseases: the orphanet database. PLoS ONE. (2017) 12:e0170365-e. 10.1371/journal.pone.0170365 28099516 PMC5242437

[18] Kremp O Dosquet P Rath A. Professional clinical guidelines for rare diseases: methodology. Orphanet J Rare Dis. (2012) 7:A12. 10.1186/1750-1172-7-S2-A1229866202

[19] Woolf SH Grol R Hutchinson A Eccles M Grimshaw J. Potential benefits, limitations, and harms of clinical guidelines. BMJ. (1999) 318:527–30. 10.1136/bmj.318.7182.527 10024268 PMC1114973

[20] Orphanet. User Satisfaction Survey of the Orphanet Website. (2015). Avilable online at: https://www.orpha.net/orphacom/cahiers/docs/GB/Orphanet_survey2015.pdf (accessed May 2021).

[21] National Institute for Health and Care Excellence. Epilepsies: Diagnosis and Management (CG137). (2021). Available online at: https://www.nice.org.uk/guidance/cg137 (accessed May 2021).

[22] Hancock E Osborne J Hancock E. Treatment of infantile spasms. Cochrane Database Syst Rev. (2002). 10.1002/14651858.CD001770 12076419

[23] Hancock EC Cross JH. Treatment of lennox-gastaut syndrome. Cochrane Database Syst Rev. (2013):Cd003277. 10.1002/14651858.CD003277.pub3 23450537 PMC7144815

[24] Cipriani A Furukawa TA Barbui C. What is a cochrane review? Epidemiol Psychiatr Sci. (2011) 20:231–3. 10.1017/S2045796011000436 21922964

[25] European Medicines Agency. Diacomit (stiripentol.) (2020). Available online at: https://www.ema.europa.eu/en/medicines/human/EPAR/diacomit (accessed May 2021).

[26] European Medicines Agency. EU/3/14/1339: Cannabidiol. (2019). Available online at: https://www.ema.europa.eu/en/medicines/human/orphan-designations/eu3141339 (accessed May 2021).

[27] Food and Drug Administration. Diacomit (Stiripentol): Highlights Of Prescribing Information. (2018). Available online at: https://www.accessdata.fda.gov/drugsatfda_docs/label/2018/206709s000,207223s000lbl.pdf (accessed May 2021).

[28] Food and Drug Administration. EPIDIOLEX® (Cannabidiol) oral solution: Highlights of prescribing information. (2018). Available online at: https://www.accessdata.fda.gov/drugsatfda_docs/label/2020/210365s005s006s007lbl.pdf (assessed May 2021).

[29] National Institute for Health and Care Excellence. Stiripentol. (2020). Available online at https://bnfc.nice.org.uk/drug/stiripentol.html (accessed May 2021).

[30] Food and Drug Administration. BANZEL® (Rufinamide): Highlights of Prescribing Information. (2015). Available online at: https://www.accessdata.fda.gov/drugsatfda_docs/label/2015/021911s012lbl.pdf (assessed May 2021).

[31] Food and Drug Administration. LAMICTAL (Lamotrigine): Highlights of Prescribing Information. (2020). Available online at: https://www.accessdata.fda.gov/drugsatfda_docs/label/2020/020241s058,020764s051,022251s022lbl.pdf (accessed May 2021).

[32] Food and Drug Administration. Topamax (Topiramate): Highlights Of Prescribing Information. (2014). Available online at: https://www.accessdata.fda.gov/drugsatfda_docs/label/2014/020505s055,020844s046lbl.pdf (accessed May 2021)

[33] Food and Drug Administration. SYMPAZANTM (Clobazam): Highlights of Prescribing Information. (2018). Available online at: https://www.accessdata.fda.gov/drugsatfda_docs/label/2018/210833s000lbl.pdf (accessed May 2021).

[34] Food and Drug Administration. FELBATOL® (Felbamate): FDA Approved Labeling Text. (2012). Available online at: https://www.accessdata.fda.gov/drugsatfda_docs/label/2012/020189s027lbl.pdf (accessed May 2021).

[35] Lim Z Wong K Olson HE Bergin AM Downs J Leonard H. Use of the ketogenic diet to manage refractory epilepsy in CDKL5 disorder: experience of >100 patients. Epilepsia. (2017) 58:1415–22. 10.1111/epi.13813 28605011

[36] Wheless JW Clarke DF Arzimanoglou A Carpenter D. Treatment of pediatric epilepsy: European expert opinion, 2007. Epileptic Disord. (2007) 9:353–412. 10.1684/epd.2007.0144 18077226

[37] Wilmshurst JM Gaillard WD Vinayan KP Tsuchida TN Plouin P Van Bogaert P . Summary of recommendations for the management of infantile seizures: task force report for the ILAE commission of pediatrics. Epilepsia. (2015) 56:1185–97. 10.1111/epi.13057 26122601

[38] Wheless JW Clarke DF Carpenter D. Treatment of pediatric epilepsy: expert opinion, 2005. J Child Neurol. (2005) 20:S1–56. 10.1177/088307380502000101 16615562

[39] Scottish Intercollegiate Guidelines Network. Diagnosis And Management Of Epilepsy In Adults. (2018). Available online at: https://www.sign.ac.uk/media/1079/sign143_2018.pdf (accessed May 2021).

[40] Guerrini R Chiamenti G Mugelli A Ruggieri M Lubrano R Provinciali L . Linee Guida: Epilessie Pediatriche. Associazione Italiana Contro L'epilessia. (2017). Available online at: http://www.aice-epilessia.it/index.php?option=com_content&view=article&id=176:linee-guida-epilessie-in-eta-pediatrica&catid=1:banner (accessed May 2021).

[41] Auvin S Höhn S Dozières-Puyravel B Hirsch E Lesca G Marie-Conia E . National Protocol for Diagnosis and Care (NPSP) Myoclonic Epilepsy in Infants. (2019). Available online at: https://www.has-sante.fr/upload/docs/application/pdf/2019-06/pnds_texte_epilepsie_myoclonique_nourrisson_mai_2019.pdf (accessed May 2021).

[42] Epilepsy Implementation Task Force (EITF). Provincial Guidelines for the Management of Medically-Refractory Epilepsy in Adults and Children Who Are Not Candidates for Epilepsy Surgery. Critical Care Services Ontario. (2016). Available online at: https://oen.echoontario.ca/media/Prov-Guidelines-for-Management-of-MRE-in-Adults-Children-not-candidates-for-Surgery_EN.pdf (accessed May 2021).

[43] Snead C Burneo J Ribaupierre SD Elliot-Miller P. Ferguson E Gould L Clinical Guidelines for the Management of Epilepsy in Adults Children. (2020). Available online at: https://clinictocommunity.ca/wp-content/uploads/2021/01/ManagementGuidelines_Nov2020.pdf (accessed May 2021).

[44] Sánchez-Álvarez J Ruiz-Giménez J Roldán Aparicio S Serrano-Castro P Arenas Cabrera C Camino León R . Guía Andaluza de la Epilepsia 2015: Diagnóstico y tratamiento de la epilepsia en niños y adultos. Available online at: https://escueladepacientes.es/images/Pdfs/SADE%20-%20Gu%C3%ADa%20Andaluza%20de%20Epilepsia%202015.pdf (accessed May 2021).

[45] Cross HJ Auvin S Falip M Striano P Arzimanoglou A. Expert opinion on the management of lennox–gastaut syndrome: treatment algorithms and practical considerations. Front Neurol. (2017) 8:505. 10.3389/fneur.2017.00505 29085326 PMC5649136

[46] Kerr M Guidelines Working Group Scheepers M Arvio M Beavis J Brandt C . Consensus guidelines into the management of epilepsy in adults with an intellectual disability. J Intellect Disabil Res. (2009) 53:687–94. 10.1111/j.1365-2788.2009.01182.x 19527434

[47] Wirrell EC Laux L Donner E Jette N Knupp K Meskis MA . Optimizing the diagnosis and management of dravet syndrome: recommendations from a North American consensus panel. Pediatric Neurol. (2017) 68:18–34. 10.1016/j.pediatrneurol.2017.01.025 28284397

[48] Sanders DB Wolfe GI Benatar M Evoli A Gilhus NE Illa I . International consensus guidance for management of myasthenia gravis: executive summary. Neurology. (2016) 87:419–25. 10.1212/WNL.0000000000002790 27358333 PMC4977114

[49] Glauser T Ben-Menachem E Bourgeois B Cnaan A Guerreiro C Kalviainen R . Updated ILAE evidence review of antiepileptic drug efficacy and effectiveness as initial monotherapy for epileptic seizures and syndromes. Epilepsia. (2013) 54:551–63. 10.1111/epi.12074 23350722

[50] Wang Z Norris SL Bero L. The advantages and limitations of guideline adaptation frameworks. Implementation Science. (2018) 13:72. 10.1186/s13012-018-0763-4 29843737 PMC5975671

[51] Munn Z Stern C Aromataris E Lockwood C Jordan Z. What kind of systematic review should I conduct? a proposed typology and guidance for systematic reviewers in the medical and health sciences. BMC Med Res Methodol. (2018) 18:5. 10.1186/s12874-017-0468-4 29316881 PMC5761190

[52] Paul C Gourraud P-A Bronsard V Prey S Puzenat E Aractingi S . Evidence-based recommendations to assess psoriasis severity: systematic literature review and expert opinion of a panel of dermatologists. J Eur Acad Dermatol Venereol. (2010) 24:2–9. 10.1111/j.1468-3083.2009.03561.x 20443994

[53] Eubank BH Mohtadi NG Lafave MR Wiley JP Bois AJ Boorman RS . Using the modified Delphi method to establish clinical consensus for the diagnosis and treatment of patients with rotator cuff pathology. BMC Med Res Methodol. (2016) 16:56. 10.1186/s12874-016-0165-8 27206853 PMC4875724

[54] Brouwers MC Kerkvliet K Spithoff K. The AGREE reporting checklist: a tool to improve reporting of clinical practice guidelines. BMJ. (2016) 352:i1152. 10.1136/bmj.i1152 26957104 PMC5118873

[55] International League Against Epilepsy. Guidelines. (2021). Available online at: https://www.ilae.org/guidelines (accessed May 2021).

[56] National Organization for Rare Disorders. Lennox-Gastaut Syndrome. (2017). Available online at: https://rarediseases.org/rare-diseases/lennox-gastaut-syndrome/ (accessed May 2021).

[57] Richter T Janoudi G Amegatse W Nester-Parr S. Characteristics of drugs for ultra-rare diseases versus drugs for other rare diseases in HTA submissions made to the CADTH CDR. Orphanet J Rare Dis. (2018) 13:15. 10.1186/s13023-018-0762-1 29386040 PMC5793441

[58] Food and Drug Administration. FINTEPLA® (fenfluramine) Oral Solution: Highlights of Prescribing Information. (2020). Available online at: https://www.accessdata.fda.gov/drugsatfda_docs/label/2020/212102s000lbl.pdf (accessed May 2021).

[59] Brigo F Jones K Eltze C Matricardi S. Anti-seizure medications for Lennox-Gastaut syndrome. Cochrane Database Syst Rev. (2021) 4:CD003277. 10.1002/14651858.CD003277.pub4 33825230 PMC8095011

[60] Amin S Majumdar A Mallick AA Patel J Scatchard R Partridge CA . Caregiver's perception of epilepsy treatment, quality of life and comorbidities in an international cohort of CDKL5 patients. Hippokratia. (2017) 21:130–5. 10.1016/j.ejpn.2017.04.1141 30479474 PMC6247997

[61] Sharawat IK Panda PK Kasinathan A Panda P Dawman L Joshi K. Efficacy and tolerability of fenfluramine in patients with dravet syndrome: a systematic review and meta-analysis. Seizure. (2021) 85:119–26. 10.1016/j.seizure.2020.12.016 33461030

[62] Lee SK. Old versus new: why do we need new antiepileptic drugs? J Epilepsy Res. (2014) 4:39–44. 10.14581/jer.14010 25625087 PMC4295052

[63] National Institute for Health and Care Excellence. Sodium valproate. (2019). Available online at: https://bnf.nice.org.uk/drug/sodiumvalproate (accessed May 2021).

[64] National Institute for Health and Care Excellence. Topiramate. (2019). Available online at: https://bnf.nice.org.uk/drug/topiramate (accessed May 2021).

[65] National Institute for Health and Care Excellence. Zonisamide. (2019). Available online at: https://bnf.nice.org.uk/drug/zonisamide (accessed May 2021).

[66] Higgins J Thomas J Chandler J Cumpston M Li T Page M . Cochrane handbook for systematic reviews of interventions version 6.0 (updated July 2019). Cochrane. (2019). Available online at: www.training.cochrane.org/handbook

[67] Kanner AM Ashman E Gloss D Harden C Bourgeois B Bautista JF . Practice guideline update summary: efficacy and tolerability of the new antiepileptic drugs II: treatment-resistant epilepsy: report of the guideline development, dissemination, and implementation subcommittee of the American academy of neurology and the American Epilepsy Society. Neurology. (2018) 91:82–90. 10.1212/WNL.0000000000005756 29898974

