# Supplementary material for: Treatment Guidelines for Rare, Early-Onset, Treatment-Resistant Epileptic Conditions: A Literature Review on Dravet Syndrome, Lennox-Gastaut Syndrome and CDKL5 Deficiency Disorder
Source: Front Neurol. 2021 Oct 25;12:734612. doi: 10.3389/fneur.2021.734612 (PMC8573384; doi:10.3389/fneur.2021.734612)
Supplement: Supplementary file 1 [file Table_1.DOCX]

Table S1. Information sources and search strategies

| Information source | URL | Search date(s) | Search strategy ^a^ |
| --- | --- | --- | --- |
| Guideline Central | [https://www.guidelinecentral.com](https://www.guidelinecentral.com/) | 28^th^ January 2021 | The webpage was searched for the following terms (using the general search function):  *“dravet", “SMEI”, “severe myoclonus epilepsy of infancy”, “severe myoclonic epilepsy of infancy”, “lennox gastaut”, “cdkl5 deficiency”, “CDD”* |
| National Organization for Rare Disorders (NORD) | [https://rarediseases.org](https://rarediseases.org/) | 28^th^–29^th^ January 2021 | The webpage was searched for the following terms (using the advanced search function, searching ‘Rare Disease Database’ and ‘Physician Guides’ categories):  *"dravet", “SMEI”, “severe myoclonus epilepsy of infancy”, “severe myoclonic epilepsy of infancy”, “lennox gastaut”, “cdkl5 deficiency”,* *“CDD”* |
| International League Against Epilepsy (ILAE) | <https://www.ilae.org> | 28^th^ January 2021 | The webpage was searched for the following terms (using the general search function):  *"dravet", “SMEI”, “severe myoclonus epilepsy of infancy”, “severe myoclonic epilepsy of infancy”, “lennox gastaut”, “cdkl5 deficiency”*, *“CDD”* |
| American Academy of Neurology (AAN) | <https://www.aan.com> | 25^th^ January 2021 | The webpage was searched for the following terms (using the general search function):  *“dravet", “SMEI”, “severe myoclonus epilepsy of infancy”, “severe myoclonic epilepsy of infancy”, “lennox gastaut”, “cdkl5 deficiency”, “CDD”* |
| American Epilepsy Society (AES) | <https://www.aesnet.org> | 1^st^ February 2021 | The webpage was searched for the following terms (using the general search function):  *“dravet", “SMEI”, “severe myoclonus epilepsy of infancy”, “severe myoclonic epilepsy of infancy”, “lennox gastaut”, “cdkl5 deficiency”, “CDD”* |
| Orphanet | [https://www.orpha.net](https://www.orpha.net/consor/cgi-bin/Disease_Search.php?lng=EN) | 28^th^ January 2021 | The webpage was searched for the following terms (using the “Disease name” setting):  *"dravet", “SMEI”, “severe myoclonus epilepsy of infancy”, “severe myoclonic epilepsy of infancy”, “lennox gastaut”, “cdkl5 deficiency”*, *“CDD”* |
| Google | [www.google.com](http://www.google.com) | 10^th^–17^th^ February 2021 | *"dravet", “SMEI”, “severe myoclonus epilepsy of infancy”, “severe myoclonic epilepsy of infancy”, “lennox gastaut”, “cdkl5 deficiency”, “CDD”* AND *“guideline” or “guidance”* AND *“United Kingdom”, “UK”, “Australia”, “Canada,” “US”, “United States”, “America”, “American” or “international”* |
|  | [www.google.es](http://www.google.es) | 11^th^–15^th^ February 2021 | *"dravet", “Epilepsia Mioclónica Grave de la Infancia”, “lennox gastaut”, or “cdkl5”* AND *“epilepsia directrices guia” or “directrices guia” or “pauta” or “recomendacion”* AND *“españa”* |
|  | [www.google.it](http://www.google.it) | 15^th^ February 2021 | *"Sindrome di Dravet", “Epilessia mioclonica grave neonatale”, “Epilessia neonatale grave con crisi miocloniche”, “SMEI”, “Sindrome di Lennox-Gastaut”,* or *“cdkl5”* AND *“guida” or “Linee guida”* AND *“Italia”* |
|  | [www.google.fr](http://www.google.fr) | 18^th^ February 2021 | *"Syndrome de Dravet", “Epilepsie myoclonique sévère du nourrisson”, “EMSN”, “Syndrome de Lennox-Gastaut”, “SLG”, “Encéphalopathie épileptique associée à cdkl5”* or *“deficit cdkl5”* AND *“conseil” or “lignes directrice”* AND *“France”* |
|  | [www.google.de](http://www.google.de) | 16^th^ February 2021 | *"dravet", “schwere frühkindliche myoklonische Epilepsie”, “lennox gastaut”,“cdkl5”*, *“CDD”* AND *“richtlinie” or “leitlinie”* AND *“Deutschland”* |
|  | [www.google.ch](http://www.google.ch) | 16^th^ February 2021 | *"dravet", “schwere frühkindliche myoklonische Epilepsie”, “lennox gastaut”,“cdkl5”*, *“CDD”* AND *“richtlinie” or “leitlinie”* AND *“Schweiz”* |
|  | [www.google.il](http://www.google.il) | 11^th^–15^th^ February 2021 | *"dravet", “SMEI”, “severe myoclonus epilepsy of infancy”, “severe myoclonic epilepsy of infancy”, “lennox gastaut”, “cdkl5 deficiency” or “CDD”* AND “*guideline” or “guidance*” AND “*Israel*” |
|  |  |  | תסמונת דרווה  אפילפסיה מיוקלונית חמורה בינקות  תסמונת לנוקס-גסטו  AND  קו מנחה  הדרכה  AND  ישראל |
|  | [www.google.jp](http://www.google.jp) | 16^th^ February 2021 | (Dravet症候群), (ドラベ症候群), (乳児重症ミオクロニーてんかん), (レノックス・ガストー), (レンノックス・ガストー), (Lennox-Gastaut 症候群), (CDD) or (cdkl5欠乏症) AND (ガイドライン, ガイド), (指導), or (指針) AND (アドバイス) |
| National HTA Bodies/Medicine Agencies for Countries of Interest | | | |
| National Institute for Health and Care Excellence (NICE) | [https://www.nice.org.uk](https://www.nice.org.uk/) | 25^th^ January 2021 | The webpage was searched for the following terms (using the general search function):  *"dravet", “SMEI”, “severe myoclonus epilepsy of infancy”, “severe myoclonic epilepsy of infancy”, “lennox gastaut”, “cdkl5 deficiency”* and *“CDD”* |
| Pharmaceutical Benefits Scheme (PBS) | <http://www.pbs.gov.au/pbs/home> | 26^th^ January 2021 | The webpage was searched for the following terms (using the general search function):  *"dravet", “SMEI”, “severe myoclonus epilepsy of infancy”, “severe myoclonic epilepsy of infancy”, “lennox gastaut”, “cdkl5 deficiency”* and *“CDD”* |
| Canadian Agency for Drugs and Technologies in Health (CADTH) | [https://cadth.ca](https://cadth.ca/) | 29^th^ January 2021 | The webpage was searched for the following terms (using the general search function):  *"dravet", “SMEI”, “severe myoclonus epilepsy of infancy”, “severe myoclonic epilepsy of infancy”, “lennox gastaut”, “cdkl5 deficiency”*, *“CDD.* |
| Ministerio de Sanidad, Consumo y Bienestar Social (MSCBS) | <http://www.mscbs.gob.es/home.htm> | 1^st^ February 2021 | The webpage was searched for the following terms (using the general search function):  *"dravet", “Síndrome de Dravet”, “Severe myoclonic epilepsy of infancy”, “severe myoclonic epilepsy of infancy”, “Epilepsia Mioclónica Grave de la Infancia”, “"Epilepsia Mioclónica Severa de la Infancia", “EMGI”, “lennox gastaut”, “cdkl5”* |
| Agenzia Italiana del Farmaco (AIFA) | <http://www.agenziafarmaco.gov.it> | 1^st^ February 2021 | The webpage was searched for the following terms (using the general search function):  *"Sindrome di Dravet", “Epilessia mioclonica grave neonatale”, “Epilessia neonatale grave con crisi miocloniche”, “SMEI”, “Sindrome di Lennox-Gastaut”* and *“cdkl5”* |
| Haute Autorité de Santé (HAS) | [https://www.has-sante.fr/portail](https://www.has-sante.fr/portail/) | 29^th^ January 2021 | The webpage was searched for the following terms (using the general search function):  *"Syndrome de Dravet", “Epilepsie myoclonique sévère du nourrisson”, “EMSN”, “Syndrome de Lennox-Gastaut”, “SLG”, “Encéphalopathie épileptique associée à cdkl5”* and *“déficit cdkl5”* |
| Gemeinsamer Bundesausschuss (G-BA) | [https://www.g-ba.de](https://www.g-ba.de/) | 29^th^ January 2021 | The webpage was searched for the following terms (using the general search function):  *"dravet", “severe myoclonus epilepsy of infancy”, “schwere frühkindliche myoklonische Epilepsie”, “lennox gastaut”, “cdkl5”* and *“CDD”* |
| Bundesamt für Gesundheit (BAG) | <https://www.bag.admin.ch/bag/de/home.html> | 29^th^ January 2021 | The webpage was searched for the following terms (using the general search function):  *"dravet", “severe myoclonus epilepsy of infancy”, “schwere frühkindliche myoklonische Epilepsie”, “lennox gastaut”, “cdkl5”* and *“CDD”* |
| State of Israel – Ministry of Health | <https://www.health.gov.il/English/Pages/HomePage.aspx> | 2^nd^ February 2021 | The webpage was searched for the following terms (using the general search function):  *”dravet", “SMEI”, “severe myoclonus epilepsy of infancy”, “severe myoclonic epilepsy of infancy”, “lennox gastaut”, “cdkl5 deficiency”* and *“CDD”*  The webpage was also searched in Hebrew using the following terms:  תסמונת דרווה  אפילפסיה מיוקלונית חמורה בינקות  תסמונת לנוקס-גסטו  קו מנחה  הדרכה  ישראל |
| Ministry of Health, Labour and Welfare (MHLW) | [https://www.mhlw.go.jp/english](https://www.mhlw.go.jp/english/) | 3^rd^ February 2021 | The webpage was searched for the following terms (using the general search function):  *(dravet), (SMEI), (severe myoclonus epilepsy of infancy), (severe myoclonic epilepsy of infancy), (lennox gastaut), (cdkl5 deficiency)* and *(CDD)*  The webpage was also searched in Japanese using the following terms:  (Dravet症候群), (ドラベ症候群), (乳児重症ミオクロニーてんかん), (レノックス・ガストー), (レンノックス・ガストー), (Lennox-Gastaut 症候群), (CDD), (cdkl5欠乏症), (ガイドライン, ガイド), (指導), (指針), (アドバイス) |
| Institute for Clinical and Economic Review (ICER) | https://icer-review.org/ | 25^th^ January 2021 | The webpage was searched for the following terms (using the general search function):  *“dravet", “SMEI”, “severe myoclonus epilepsy of infancy”, “severe myoclonic epilepsy of infancy”, “lennox gastaut”, “cdkl5 deficiency”, “CDD”* |

All results were screened for relevance, except for Google searches, where the first 3 pages of search results for each search were screened for relevance.

AAN, American Academy of Neurology; AES, American Epilepsy Society; AIFA, Agenzia Italiana del Farmico; BAG, Bundesamt für Gesundheit; CADTH, Canadian Agency for Drugs and Technologies in Health; CDD, CDKL5 deficiency disorder; EMSN, Epilepsie myoclonique sévère du nourrisson; G-BA, Gemeinsamer Bundesausschuss; HAS, Haute Autorité de Santé; ICER, Institute for Clinical and Economic Review; HTA, health technology assessment; ILAE, International League Against Epilepsy; MHLW, Ministry of Health, Labour and Welfare; MSCBS, Ministerio de Sanidad, Consumo y Bienestar Social; NICE, National Institute for Health and Care Excellence; NORD, National Organisation for Rare Disorders; PBS, Pharmaceutical Benefits Scheme; SLG, Syndrome de Lennox-Gastaut; SMEI, severe myoclonic epilepsy of infancy; UK, United Kingdom; US, United States.

Table S2. List of included guidelines

| Title | Developing bodies | Region | Date (latest revision) | Indication(s) | Reference |
| --- | --- | --- | --- | --- | --- |
| Epilepsies: the diagnosis and management of epilepsies in adults and children in primary and secondary care | NICE | UK | January 2021 | - Dravet syndrome - Lennox-Gastaut syndrome | NICE, 2021[[21](#_ENREF_21)] |
| Provincial Guidelines for the Management of Medically-Refractory Epilepsy in Adults and Children Who Are Not Candidates for Epilepsy Surgery | EITF, Critical Care Services Ontario and Provincial Neurosurgery Ontario | Canada (Ontario) | March 2016 | - Dravet syndrome - Lennox-Gastaut syndrome | EITF, 2016[[42](#_ENREF_42)] |
| Clinical Guidelines for the Management of Epilepsy in Adults and Children | EITF | Canada (Ontario) | March 2020 | - Dravet syndrome - Lennox-Gastaut syndrome | Snead C. et al. 2020[[43](#_ENREF_43)] |
| Expert Opinion on the Management of Lennox-Gastaut Syndrome: Treatment Algorithms and Practical Considerations | NR | International | September 2017 | - Lennox-Gastaut syndrome | Cross H. et al. 2017[[45](#_ENREF_45)] |
| Epilepsies in Children and Young People: Investigative Procedures and Management | SIGN | UK (Scotland) | November 2018 | - Dravet syndrome - Lennox-Gastaut syndrome | SIGN, 2018[[68](#_ENREF_68)] |
| Diagnosis and management of epilepsy in adults | SIGN | UK (Scotland) | September 2018 | - Lennox-Gastaut syndrome | SIGN, 2018[[39](#_ENREF_39)] |
| Guideline for Management of Children with Epileptic Seizures in British Columbia | Division of Neurology, British Columbia Children's Hospital (in collaboration with the Departments of Paediatrics and Psychology and the British Columbia Paediatric Society) | Canada (British Columbia) | April 2011 | - Dravet syndrome | Farrell K., Connolly M. 2011[[69](#_ENREF_69)] |
| ILAE Lecture Notes: From Channels to Commissioning - A Practical Guide to Epilepsy; Chapter 30 - Drug Treatment of Paediatric Epilepsy | ILAE (UK Chapter) and Epilepsy Society; sponsored by UCB Pharma and Eisai | UK | 2017 (15th edition; originally published 1987) | - Dravet syndrome - Lennox-Gastaut syndrome | Appleton R.E. and Cross H., 2017[[70](#_ENREF_70)] |
| Guidance on the Use of Cannabis-Based Products for Medicinal Use in Children and Young People with Epilepsy | NHS England (on behalf on the devolved nations). Approved by Association of British Neurologist and the ILAE (UK Chapter). | UK (England) | October 2018 | - Dravet syndrome - Lennox-Gastaut syndrome | BPNA, 2018[[71](#_ENREF_71)] |
| Guidance for the Use of Medicinal Cannabis in the Treatment of Epilepsy in Paediatric and Young Adult Patients in Australia | Australian Government Department of Health | Australia | December 2017 | - Dravet syndrome - Lennox-Gastaut syndrome | TGA, 2017[[72](#_ENREF_72)] |
| DIACOMIT (stiripentol): Transparency Committee Opinion | HAS | France | June 2007 | - Dravet syndrome | HAS, 2007[[73](#_ENREF_73)] |
| INOVELON (rufinamide): Transparency Committee Opinion | HAS | France | February 2009 | - Lennox-Gastaut syndrome | HAS, 2009[[74](#_ENREF_74)] |
| LIKOZAM (clobazam): Transparency Committee Opinion | HAS | France | September 2016 | - Lennox-Gastaut syndrome | HAS, 2016[[75](#_ENREF_75)] |
| ZONEGRAN (zonisamide): Transparency Committee Opinion | HAS | France | December 2014 | - Lennox-Gastaut syndrome | HAS, 2014[[76](#_ENREF_76)] |
| Guide - Chronic Diseases: Severe Epilepsies | HAS | France | July 2007 | - Dravet syndrome - Lennox-Gastaut syndrome | Beuzon S. et al. 2007[[77](#_ENREF_77)] |
| Epilepsy: Care of children and adults | HAS | France | October 2020 | - Dravet syndrome - Lennox-Gastaut syndrome | HAS, 2020[[78](#_ENREF_78)] |
| National Protocol for Diagnosis and Care (NPSP) Myoclonic Epilepsy in Infants | Reference Centre for Rare Epilepsies of the Robert-Debré Hospital (APHP) | France | May 2019 | - Dravet syndrome | Auvin S. et al. 2019[[41](#_ENREF_41)] |
| First epileptic seizure and epilepsies in adulthood | DGN | Germany | April 2017 | - Lennox-Gastaut syndrome | Elger C. et al. 2017[[79](#_ENREF_79)] |
| Lennox-Gastaut Syndrome | Deutsche Gesellschaft für Epileptologie | Germany | April 2008 | - Lennox-Gastaut syndrome | Borusiak P. and Boenigk H.E., 2008[[80](#_ENREF_80)] |
| Treatment of Epilepsy in Children | Società Italiana di Neuropsichiatria dell'Infazia e dell'Adolescenza (SINPIA) and University Hospital (AOU) Meyer | Italy | January 2017 | - Dravet syndrome - Lennox-Gastaut syndrome | Guerrini R. et al. 2017[[40](#_ENREF_40)] |
| Diagnosis and Treatment of Epilepsies: Guideline for the Tuscany Region (SNLG) | Tuscany Regional Health Council | Italy (Tuscany) | 2014 (Revised version of the 2006 original) | - Dravet syndrome - Lennox-Gastaut syndrome | Campostrini, R. et al. 2014[[81](#_ENREF_81)] |
| Updated Practical Manual for the Diagnosis and Treatment of Epilepsy | NR | Italy | July 2012 | - Dravet syndrome - Lennox-Gastaut syndrome | Anzellotti F. and Onofrj M., 2012[[82](#_ENREF_82)] |
| Epilepsy Management Guideline | Japan Neurology Society | Japan | 2018 | - Dravet syndrome - Lennox-Gastaut syndrome | Ugawa Y. et al. 2018[[83](#_ENREF_83)] |
| Tuberous Sclerosis Complex Diagnosis Criteria and Treatment Guideline: Revised Version | Japan Tuberous Sclerosis Complex Society and Refractory Disease Policy Study Group formed by Ministry of Health, Labour and Welfare and Japanese Dermatological Association | Japan | 2018 | - Lennox-Gastaut syndrome | Kaneda M. et al. 2018[[84](#_ENREF_84)] |
| Childhood Epilepsy Medical Treatment Guide Understood by Flow Chart | Okayama University | Japan | 2011 | - Dravet syndrome - Lennox-Gastaut syndrome | Ohtsuka Y. et al. 2011[[85](#_ENREF_85)] |
| Drug Treatment Guidelines for Epilepsy Using New Anti-Epileptic Drugs | The Japan Epilepsy Society | Japan | 2010 | - Dravet syndrome - Lennox-Gastaut syndrome | Ikeda A. et al. 2010[[86](#_ENREF_86)] |
| Textbook for Epilepsy – New Version | NR | Japan | 2012 | - Dravet syndrome - Lennox-Gastaut syndrome | Tsuji S. and Ugawa Y., 2012[[87](#_ENREF_87)] |
| Therapeutic Information of the National Health System: New Active Ingredients (2010 Review) | MSCBS | Spain | 2011, reporting on 2010 | - Dravet syndrome | MSCBS, 2011[[88](#_ENREF_88)] |
| Guidelines for Seizure Management in Palliative Care: Proposal for an Updated Clinical Practise Model Based on a Systematic Literature Review | Clínica San Vicente (neurorehabilitation clinic) | Spain | November 2016 | - Dravet syndrome - Lennox-Gastaut syndrome | León Ruiz M. et al. 2016[[89](#_ENREF_89)] |
| Drug Resistant Epilepsy: Definition and Treatment Alternatives | NR | Spain | June 2014 | - Dravet syndrome - Lennox-Gastaut syndrome | López González F.J. et al. 2014[[90](#_ENREF_90)] |
| Combined Treatment with Anti-Epileptic Drugs: Andalusian Epilepsy Guide 2015 | Andalucian Epilepsy Society (SAdE) | Spain (Andalusia) | 2015 | - Dravet syndrome - Lennox-Gastaut syndrome | Sánchez-Álvarez J.C. et al. 2015[[91](#_ENREF_91)] |
| General Advice of Official Pharmacy Colleges: Pharmacological Point No. 95, Epilepsy | Consejo General de Colegios Oficiales de Farmacéuticos | Spain | April 2015 | - Lennox-Gastaut syndrome | Consejo General de Colegios Oficiales de Farmacéuticos, 2015[[92](#_ENREF_92)] |
| Andalusian Epilepsy Guide 2015: Diagnosis and Treatment of Epilepsy in Children and Adults | Andalusian Epilepsy Society | Spain (Andalusia) | 2015 | - Dravet syndrome - Lennox-Gastaut syndrome | Sánchez-Álvarez J.C. et al. 2015[[44](#_ENREF_44)] |
| Diagnostic and therapeutic recommendations of the SEN 2019 | Sociedad Española de Neurologia | Spain | 2019 | - Dravet syndrome - Lennox-Gastaut syndrome | López González F.J. et al. 2019[[93](#_ENREF_93)] |
| Optimizing the Diagnosis and Management of Dravet Syndrome: Recommendations from a North American Consensus Panel | Pediatric Epilepsy Research Consortium, Dravet Syndrome Foundation and Intractable Childhood Epilepsy Alliance | International  (US and Canada) | January 2017 | - Dravet syndrome | Wirrell E.C. et al. 2017[[47](#_ENREF_47)] |
| Practice guideline update summary: Efficacy and tolerability of the new antiepileptic drugs II: Treatment-resistant epilepsy | American Academy of Neurology (AAN), American Epilepsy Society | US | July 2018 | - Lennox-Gastaut syndrome | Kanner A.M. et al. 2018[[67](#_ENREF_67)] |
| Consensus guidelines in the management of epilepsy in adults with an intellectual disability | International Society for the Scientific Study of Intellectual Disability (IASSID) | International | August 2009 | - Lennox-Gastaut syndrome | Kerr M. et al.  2009[[46](#_ENREF_46)] |
| Treatment of pediatric epilepsy: European expert opinion, 2007 | NR | International  (Europe) | December 2007 | - Lennox-Gastaut syndrome | Wheless J.W. et al. 2007[[36](#_ENREF_36)] |
| Summary of recommendations for the management of infantile seizures: Task Force Report for the ILAE Commission of Pediatrics | ILAE, Commission for Pediatrics | International | June 2015 | - Dravet syndrome | Wilmshurst J.M. et al. 2015[[37](#_ENREF_37)] |
| Treatment of Pediatric Epilepsy: Expert Opinion, 2005 | NR | US | November 2005 | - Lennox-Gastaut syndrome | Wheless J.W. et al. 2005[[38](#_ENREF_38)] |

AOU: Azienda Ospedaliera Universitaria; AAN: American Academy of Neurology; BPNA, British Paediatric Neurology Association; DGN, Deutsche Gesellschaft für Neurologie; EITF, Epilepsy Implementation Task Force; HAS, Haute Autorité de Santé; ILAE, International League Against Epilepsy; MSCBS, Ministerio de Sanidad, Consumo y Bienestar Social; NHS, National Health Service; NICE, National Institute for Health and Care Excellence; NPSP, National Protocol for Diagnosis and Care; NR, not reported; SIGN, Scottish Intercollegiate Guidelines Network; SINPIA, Società Italiana di Neuropsichiatria dell'Infazia e dell'Adolescenza; SNLG: Sistema nazionale linee guida; TGA, Therapeutic Goods Administration; UK, United Kingdom; US, United States.

Table S3. Treatment line-specific recommendations for DS

| **Treatment line-specific recommendations (positive)** | | |  |
| --- | --- | --- | --- |
| **First-line** | **18** | **Second-line** | **19** |
| Sodium valproate | 10 | Clobazam | 4 |
| Topiramate | 5 | Stiripentol | 3 |
| Stiripentol | 2 | Clonazepam | 2 |
| Clobazam | 1 | Ethosuximide | 2 |
|  |  | Topiramate | 2 |
|  |  | Clorazepate | 1 |
|  |  | Phenobarbital | 1 |
|  |  | Potassium bromide | 1 |
|  |  | Zonisamide | 1 |
|  |  | Levetiracetam | 1 |
|  |  | Lamotrigine | 1 |
|  |  |  |  |
| **Treatment line-specific recommendations (negative)** | | |  |
| **First-line** | **0** | **Second-line** | **8** |
| N/A |  | Carbamazepine | 1 |
|  |  | Gabapentin | 1 |
|  |  | Lamotrigine | 1 |
|  |  | Oxcarbazepine | 1 |
|  |  | Phenytoin | 1 |
|  |  | Pregabalin | 1 |
|  |  | Tiagabine | 1 |
|  |  | Vigabatrin | 1 |

DS, Dravet syndrome; N/A, not applicable.

Table S4. Treatment line-specific recommendations for LGS

| **Treatment line-specific recommendations (positive)** | | |  |
| --- | --- | --- | --- |
| **First-line** | **28** | **Second-line** | **35** |
| Sodium valproate | 14 | Lamotrigine | 9 |
| Topiramate | 3 | Topiramate | 6 |
| Rufinamide | 3 | Zonisamide | 5 |
| Clobazam | 2 | Levetiracetam | 3 |
| Lamotrigine | 2 | Rufinamide | 3 |
| Benzodiazepines | 1 | Clobazam | 2 |
| Clonazepam | 1 | Felbamate | 2 |
| Levetiracetam | 1 | Sodium valproate | 2 |
| Zonisamide | 1 | Clonazepam | 1 |
|  |  | Ethosuximide | 1 |
|  |  | Nitrazepam | 1 |
|  |  |  |  |
| **Treatment line-specific recommendations (negative)** | | |  |
| **First-line** | **0** | **Second-line** | **6** |
| N/A |  | Carbamazepine | 1 |
|  |  | Gabapentin | 1 |
|  |  | Oxcarbazepine | 1 |
|  |  | Pregabalin | 1 |
|  |  | Tiagabine | 1 |
|  |  | Vigabatrin | 1 |

LGS, Lennox-Gastaut syndrome; N/A, not applicable.
